# Hyperinflammatory State and Low T1 Adaptive Immune Response in Severe and Critical Acute COVID-19 Patients

**DOI:** 10.3389/fmed.2022.828678

**Published:** 2022-03-29

**Authors:** Mercedes Garcia-Gasalla, María Berman-Riu, Jaime Pons, Adrián Rodríguez, Amanda Iglesias, Natalia Martínez-Pomar, Isabel Llompart-Alabern, Melchor Riera, Adrián Ferré Beltrán, Albert Figueras-Castilla, Javier Murillas, Joana M. Ferrer

**Affiliations:** ^1^Department of Internal Medicine, Hospital Universitari Son Espases, Palma, Spain; ^2^Balearic Islands Health Research Institute (IdISBa), Palma, Spain; ^3^Department of Immunology, Hospital Universitari Son Espases, Palma, Spain; ^4^Centro de Investigación Biomedica en Red (CIBER) de Enfermedades Respiratorias, Hospital Universitari Son Espases, Palma, Spain; ^5^Department of Internal Medicine, Hospital Universitari Son Llàtzer, Palma, Spain; ^6^Análisis Clínicos, Hospital Universitari Son Espases, Palma, Spain; ^7^Intensive Care Unit, Hospital Universitari Son Espases, Palma, Spain

**Keywords:** COVID-19 severity, IL-18, sIL-2rα, IL-1Ra, activated memory T cell, EMRA phenotype, T regulatory cell, T1 cells

## Abstract

**Background:**

A better understanding of COVID-19 immunopathology is needed to identify the most vulnerable patients and improve treatment options.

**Objective:**

We aimed to identify immune system cell populations, cytokines, and inflammatory markers related to severity in COVID-19.

**Methods:**

139 hospitalized patients with COVID-19−58 mild/moderate and 81 severe/critical—and 74 recovered patients were included in a prospective longitudinal study. Clinical data and blood samples were obtained on admission for laboratory markers, cytokines, and lymphocyte subsets study. In the recovered patients, lymphocyte subsets were analyzed 8–12 weeks after discharge.

**Results:**

A National Early Warning Score 2 >2 (OR:41.4; CI:10.38–167.0), ferritin >583 pg/mL (OR:16.3; CI: 3.88–69.9), neutrophil/lymphocyte ratio >3 (OR: 3.5; CI: 1.08–12.0), sIL-2rα (sCD25) >512 pg/mL (OR: 3.3; CI: 1.48–7.9), IL-1Ra >94 pg/mL (OR: 3.2; IC: 1.4–7.3), and IL-18 >125 pg/mL (OR: 2.4; CI: 1.1–5.0) were associated with severe/critical COVID-19 in the multivariate models used. Lower absolute values of CD3, CD4, CD8, and CD19 lymphocytes together with higher frequencies of NK cells, a CD4 and CD8 activated (CD38+HLA-DR+) memory T cell and effector memory CD45RA+ (EMRA) phenotype, and lower T regulatory cell frequencies were found in severe/critical patients relative to mild/moderate and recovered COVID-19 patients. A significant reduction in Th1, Tfh1, and Tc1 with higher Th2, Tfh2, Tc2, and plasma cell frequencies was found in the most severe cases.

**Conclusion:**

A characteristic hyperinflammatory state with significantly elevated neutrophil/lymphocyte ratio and ferritin, IL-1Ra, sIL-2rα, and IL-18 levels together with a “low T1 lymphocyte signature” was found in severe/critical COVID-19 patients.

## Introduction

Coronavirus disease 2019 (COVID-19), caused by the severe acute respiratory syndrome coronavirus 2 (SARS-CoV-2), has been afflicting humanity since it was first described in Wuhan City, China, in December 2019 (1, 2). As of 27 October 2021, there have been 244.737.391 confirmed cases worldwide (3). Clinical presentation of COVID-19 ranges from asymptomatic cases to fatal disease. Most patients (80%) display an asymptomatic or moderate disease, but others can develop a respiratory illness requiring hospital care. Pulmonary disease can progress to acute respiratory distress syndrome (ARDS) (4, 5). Several risk factors like age, sex, and comorbidities (6), and various aspects of the immune response to SARS-CoV-2 have been related to disease severity (7). Despite great advances in the understanding of COVID-19 over the last year and a half, we are far from knowing the importance of the different components of the innate and adaptive immune system in the strength and durability of the immune response to SARS-CoV-2 and the degree of severity of the disease, and even the possible implications of the immune response in the long-COVID-19 immunopathology. Improving our understanding of the immunopathology of COVID-19 is urgently needed to identify the most vulnerable patients and to develop successful treatments and vaccines.

The immune response to SARS-CoV-2 in non-severe patients is similar to other antiviral responses. A transient innate immune response occurs followed by an adaptive immune response with an increase in T follicular helper (Tfh) cells and specific B cells, and activation of T helper (Th)1 CD4 T cells and cytotoxic CD8 T cells (8–10). Antibodies to SARS-CoV-2 are generated and response contracts as the disease resolves. However, hyperactivity of the innate immune system with hyperinflammation and deregulation of the adaptive immune response is present in critical patients (7, 11). There is great interest in finding clinical and laboratory parameters to predict the development of critical symptoms in newly diagnosed patients (12), as reflected in several attempts to develop a COVID-19 score to aid in clinical management and treatment decisions (13, 14). Moreover, the study of the different cell populations of the immune system according to disease severity in COVID-19 patients may provide substantial information on the role of the adaptive immune response on the course of the disease (9, 15). The combination of information on these cell subpopulations with other clinical and laboratory parameters may help to promptly identify patients likely to develop severe disease and eligible for personalized treatments.

Thus, the purpose of our study was to describe the different immune system cell populations, cytokines, and other inflammatory markers found in acute COVID-19 hospitalized patients with different clinical severities and to identify laboratory parameters and cell populations associated with severe/critical disease.

## Methods

### Study Design

A prospective longitudinal study was designed. Patients admitted to Son Espases Hospital in Palma de Mallorca (Spain) between 01 August 2020 and 31 November 2020 with a positive nasopharyngeal swab RT-PCR test result for COVID-19 were enrolled in the study, and recovered patients recruited in the outpatient post-COVID-19 clinic during the same period were included exclusively in the flow-cytometry part of the study. The study was approved by the local Ethics Committee (*Comité Ético de Investigación Cl*í*nica Illes Balears* n° IB 4169/20 PI) and was performed in compliance with the Declaration of Helsinki. All participants provided a written informed consent before participation in the study.

### Patients

Clinical data were recorded from patients hospitalized with a COVID-19 diagnosis, and blood samples were obtained within the first 48 h of admission. In the recovered patient group, a blood sample to study lymphocyte subsets was obtained 8–12 weeks after being discharged. Clinical and demographic data retrieved from the participants' electronic medical records included age, gender, comorbidities, and the National Early Warning Score (NEWS)-2 (16) on admission. The severity of signs and symptoms developed during hospitalization was categorized as mild/moderate (grade 1), severe (grade 2), and critical (grade 3). Mild to moderate disease was established when the patient had symptoms without pneumonia or with mild pneumonia; severe disease was established when dyspnoea was associated with a respiratory rate ≥30/min or blood oxygen saturation <93%, or a partial pressure of arterial oxygen to fraction of inspired oxygen ratio <300, and/or lung infiltrates >50% within 24–48 h from admission; and critical disease was established for cases with respiratory failure, septic shock, and/or multiple organ dysfunction or failure (17). Patients were treated during hospitalization in accordance with international COVID-19 treatment guidelines valid during the study period. Blood samples were always obtained in the first 24–48 h of admission when some severe and critical patients had already started corticosteroids, but no anti-IL6 receptor monoclonal antibodies. The most severe category developed during hospitalization was selected to classify patients, and in all cases the highest degree of severity occurred in the first 72 h of admission.

### Hematological and Biochemical Parameters

Routine blood examinations included leukocyte, neutrophil, and lymphocyte counts (cells^*^10^3^/μL) and percentages. Serum biochemical parameters recorded were ferritin (ng/L) determined by chemiluminescence immunoassay in Architect i2000 analyser (Abbot), C-reactive protein (CRP) (mg/dL), and D-dimer (μg/L) quantified by immunoturbidimetry in Architect c16000 (Abbot) and ACL TOP 700 (Instrumentation Laboratory), respectively. We used a chemiluminescence assay (IMMULITE, Siemens, Germany) to determine serum soluble IL-2 receptor alpha (sIL-2rα or sCD25), and a human cytokine magnetic bead panel (Merck Millipore, Billerica, MA, USA) to measure levels of other cytokines associated with “cytokine storm”: IL-1β, IL-1 receptor antagonist (IL-1Ra), IL-6, IL-8, IL-17A, IL-18, IL-22, interferon gamma (IFN-γ), tumor necrosis factor alpha (TNF-α), and IL-10.

### Flow Cytometry

Cell surface marker expression was analyzed by flow cytometry using a BD FACSLyric cytometer and data analysis was performed with the FlowJo software, both from Becton-Dickinson.

A surface staining protocol was applied to study the distribution of different cell subpopulations in peripheral blood. Briefly, 50 μL of peripheral whole blood were incubated with different combinations of fluorochrome conjugated monoclonal antibodies 20 min at room temperature (25°C). Red blood cells were lysed for 10 min with 2 mL of FACS Lysing solution (Becton Dickinson) and washed with phosphate-buffered saline (PBS) before flow cytometry analysis. Combinations of the following monoclonal antibodies were used: anti-CD3-PerCPCy5.5, anti-CD4-V500, anti-CD8-APCR700, anti-CD19-PECy7, anti-CD56-FITC, anti-CD45-APCH7, anti-CD45-V500, anti-HLA-DR-V450, anti-CD25-PECy7, anti-CD127-APC, anti-CD45RA-BV605, anti-CXCR5-BB515, anti-CXCR3-APC, anti-CCR6-PE, anti-CCR7-APCR700, anti-CD27-APC, anti-IgD-V450, anti-CD38-PE, anti-CD38-PerCPCy5.5, and anti-CD24-PE, all from Becton-Dickinson.

### Statistical Analysis

Categorical variables were expressed as numbers and percentages, and continuous variables as median and interquartile range (IQR) values. Proportions for categorical variables were compared between groups using the chi-squared test. Normal distribution was studied by plotting histograms and with the Shapiro-Wilk test. The *t*-test for independent groups and the Mann–Whitney *U* test were used for the comparison of continuous normally and not normally distributed variables, respectively. Correlation between variables was evaluated using Spearman's correlation test. Non-parametric Kruskall–Wallis and Mann–Whitney *U* tests were performed, respectively, to compare the difference between the three groups of patients classified as mild/moderate, severe/critical, and recovered, and the two groups classified as mild/moderate and severe/critical. Receiver operating characteristic (ROC) curves were used to assess the prognostic value of the biological markers. A prognostic value for the development of severe/critical disease was considered when the area under the ROC curve was >0.65; the optimal cut-off value providing the best trade-off between sensitivity and specificity was selected with the Youden index. Univariate and multivariate age-adjusted logistic regression analyses were performed to explore the association between laboratory parameters and the risk of developing critical disease, using the values provided by the Youden index as cut-off points. All statistical analyses were performed using SPSS (Statistical Package for the Social Sciences) version 22.0 software (SPSS Inc.) or GraphPad Prism (version 8.0 La Jolla, Ca USA). Two-sided *P*-values of < 0.05 were considered statistically significant.

## Results

### Patient Characteristics and Classification

Two hundred and thirteen COVID-19 patients agreed to participate and were included in the study: 139 patients diagnosed and hospitalized with acute COVID-19, and 74 recovered patients. Hospitalized patients had a median age of 52.0 years (p25–p75: 43.0–64.0) and 74 (53.2%) were men. COVID-19 was considered mild/moderate in 58 (41.7%), severe in 60 (43.2%), and critical in 21 (15.1%) patients. Three (2.2%) patients died. The most commonly associated comorbidities were hypertension (33.1%), dyslipidaemia (28.1%), obesity (Body Mass Index >30, 23.7%), and cardiomyopathies (13.7%). We performed an initial analysis and comparison of the three different groups and found very similar disease trajectories between severe and critical patients (data not shown). We therefore decided to consider severe and critical patients as a single group. Severe/critical disease was significantly more frequent in men (50/74 men developed severe disease vs. 31/65 women, *P* = 0.028). Compared to patients with mild/moderate disease, patients with severe/critical COVID-19 were significantly older median age 56.0 (p25–p75: 45.0–67.0) vs. 49.0 (p25–p75: 38.0–60.5). NEWS2 score median value on admission was 0.0 (p25–p75: 0.0–0.0) in patients with mild/moderate disease and 4.0 (p25–p75: 2.0–5.0) in patients who developed severe or critical COVID-19 (*P* < 0.001). Recovered patients had a median age of 56.0 years (p25–p75: 48.0–66.0) and 36 (48.6%) were men. COVID-19 had been mild/moderate in 35 (47.3%) and severe or critical in 39 (52.7%).

### Laboratory Markers and Cytokines on Admission

Significant laboratory marker and cytokine levels on admission in mild/moderate and severe/critical patients are shown in Table 1. IFN-γ, IL-1β, IL-17, and IL-22 have been excluded, since levels were undetectable in most cases and/or no significant differences between median levels in these groups were observed. Compared with patients with mild/moderate disease, patients who developed severe/critical disease had a significant lower absolute lymphocyte count and a higher neutrophil/lymphocyte ratio (NLR) as well as high levels of ferritin, D-dimer, IL-6, IL-10, sIL-2rα (sCD25), IL-1Ra, IL-18, and complement factor C3 (Table 1).

**Table 1:** Comparison of laboratory markers and cytokine levels in mild/moderate and severe/critical COVID-19.

	**Group 1 mild/moderate (*n* = 58)**	**Group 2 and 3 severe/critical (*n* = 81)**	***P*-value**
Abs Lymph (mm^3^)	1,770 (1,255–2,325)	940 (735–1,285)	**<0.001**
Ferritin (ng/mL)	223 (78–527)	763 (397–1292)	**<0.001**
D-dimer (ng/mL)	170 (126–272)	262 (185–467)	**0.009**
NLR	1.8 (1.2–2.7)	4.5 (3.0–7.0)	**<0.001**
CRP (mg/dL)	4.2 (1.0–10.7)	9.4 (4.0–16.3)	0.138
IL6 (pg/mL)	11.2 (1.8–39.2)	25.4 (7.0–72.2)	**0.007**
IL10 (pg/mL)	15.5 (2.6–55.3)	53.6 (8.5–102.1)	**0.006**
sIL-2rα (sCD25) (pg/mL)	578 (409–778)	745 (584–965)	**0.003**
IL-1Ra (pg/mL)	106 (47.8–216)	180 (105–358)	**0.045**
IL-8 (pg/mL)	51.4 (28.9–69.9)	48.7 (25.1–79.1)	0.190
IL-18 (pg/mL)	114 (56.8–206)	179 (113–322)	**<0.001**
TNF-α (pg/mL)	23.0 (7.4–54.9)	31.5 (14.5–68.1)	0.327
Complement C3	135 (113–154)	145 (131–159)	**0.022**
Complement C4	33.0 (25.5–38.2)	36.0 (28.5–48.0)	0.180

*Median and IQR (p25–p75). COVID-19, coronavirus disease 2019; CRP, C-reactive protein; IQR, interquartile range; NLR, neutrophil/lymphocyte ratio; IL-1Ra, IL-1 receptor antagonist; TNF, tumor necrosis factor; sIL-2rα, serum IL-2 receptor alpha; C3/C4, complement factors C3 and C4. Bold values mean there is statistical significance*.

### Lower Absolute Lymphocyte Counts and Higher Natural Killer (NK) Cell Frequencies in Severe/Critical Patients Compared With Mild/Moderate and Recovered COVID-19 Patients

**Figure 1:**
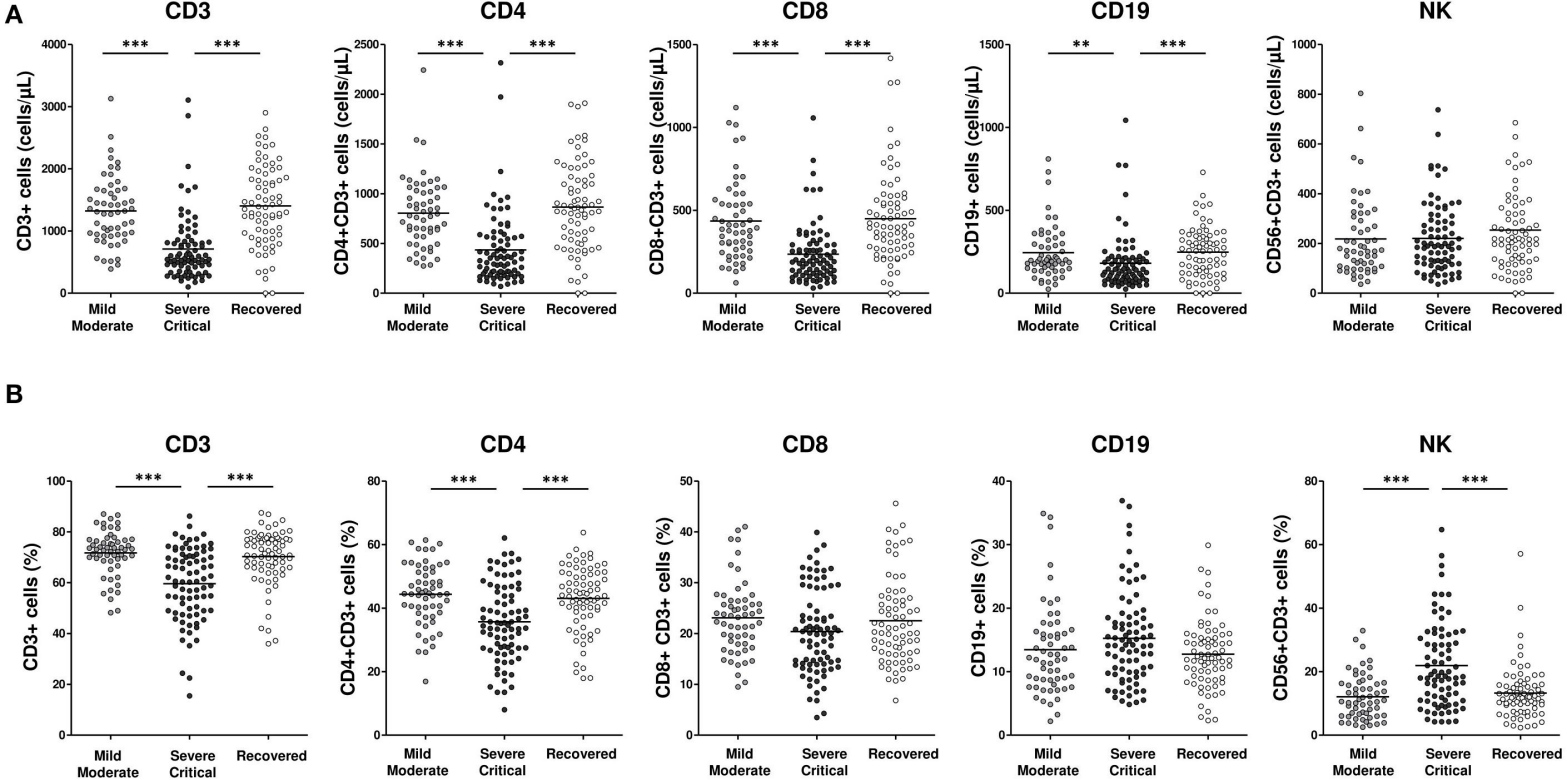
Lower lymphocytes absolute number and frequency and higher NK frequency in severe/critical COVID-19 patients. Absolute numbers **(A)** and frequency **(B)** of peripheral blood cell populations (CD3, CD4, CDS, CD19 and NK) in mild/moderate (light gray circles), severe/critical (dark gray circles) and recovered (white circles) groups of patients. Each dot represents an individual patient. Data are given as mean (Kruskal–Wallis test *P-*values: **P < 0.01; ***P < 0.001).

Lower absolute counts of CD3, CD4, CD8, and CD19 lymphocytes were detected in severe/critical patients compared with recovered (all *p*-values < 0.001) and mild/moderate patients (*p* < 0.001, *p* < 0.001, *p* < 0.001, *p* < 0.01, respectively). No differences were found in NK absolute numbers between groups (Figure 1A). These results could be a reflection of the characteristic lymphopenia observed in COVID-19 patients. To avoid the influence or bias derived from lymphopenia, the rest of the study was therefore performed considering the frequencies of the distinct subpopulations within each major population. Percentages of CD3 and CD4 cells were lower in severe/critical patients compared with mild/moderate (both *p*-values < 0.001) and recovered patients (both *p*-values < 0.001). The percentage of NK cells was higher in severe/critical patients compared with mild/moderate (both *p*-values < 0.001) and recovered patients (both *p*-values < 0.001). No differences were found in the percentages of CD8 and CD19 lymphocytes between the different groups (Figure 1B).

### Switch to an Activated (CD38+HLA-DR+) and Effector Memory CD45RA+ (EMRA) Phenotype in Severe/Critical Patients Compared With Recovered and Mild/Moderate COVID-19 Patients

**Figure 2:**
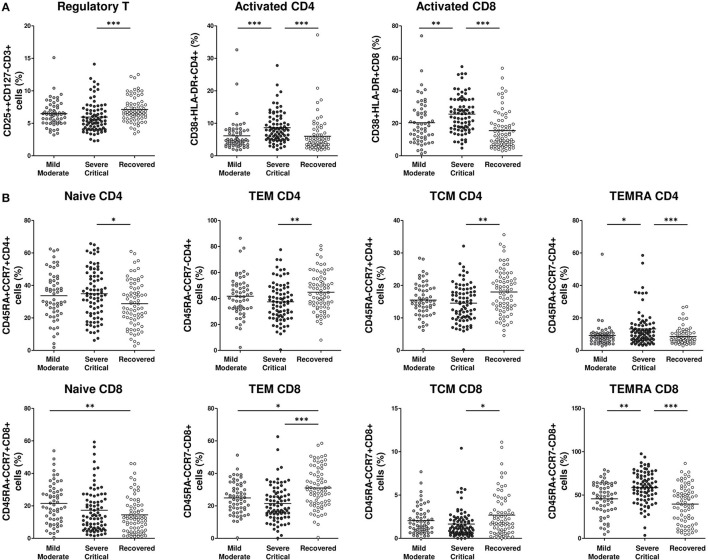
Lower regulatory T cells and higher activated (CD38+HlA-DR+) and EMRA phenotype CD4 and CDS frequency in severe/critical COVID-19 patients. Frequency of selected peripheral blood T cell populations **(A,B)** in mild/moderate (light gray circles), severe/critical (dark gray circles) and recovered (white circles) groups of patients. Each dot represents an individual patient. Data are given as mean (Kruskal–Wallis test *P-*values: *P < 0.05; **P < 0.01; ***P < 0.001). TEM, T effector memory; TCM, T central memory; EMRA, terminally differentiated effector memory cells re-expressingCD45RA.

We also evaluated the activation status and the distribution of CD4 and CD8 memory T cell subpopulations and T regulatory (Treg) cells in the three groups of patients. There was a lower Treg cell frequency in severe/critical patients compared with recovered patients (*p* < 0.001). Treg cell frequency was also lower in the mild/moderate group, but the difference was not significant (Figure 2A). The frequency of activated CD38+HLA-DR+CD4+ cells and activated CD38+HLA-DR+CD8+ cells was significantly higher in severe/critical patients compared with mild/moderate (*p* < 0.001, *p* < 0.01, respectively) and recovered patients (both *p*-values < 0.001) (Figure 2A). Naïve CD4 T cell (CD45RA+CCR7+CD4+) frequency was higher in severe/critical patients vs. recovered patients (*p* < 0.05). The frequencies of T effector memory CD4 (CD45RA-CCR7-CD4+) and CD8 (CD45RA-CCR7-CD8+) cells were lower in both mild/moderate and severe/critical patients than in recovered patients, although the differences were more pronounced for the severe/critical group (*p* < 0.01, *p* < 0.001, respectively). Similarly, severe/critical patients had fewer T central memory CD4 (CD45RA-CCR7+CD4+) and CD8 (CD45RA-CCR7+CD8+) cells than recovered patients (*p* < 0.01, *p* < 0.05, respectively). The frequency of the EMRA CD4 and CD8 phenotype characterized by effector memory T cells re-expressing CD45RA was significantly higher in severe/critical patients compared with recovered (both *p*-values < 0.001) and mild/moderate patient groups (*p* < 0.05, *p* < 0,01, respectively) (Figure 2B).

### Severe/Critical COVID-19 Patients Showed Higher Tfh2 and Th2 Cell Subpopulation Frequencies and Lower Tfh17.1, Tfh1, Th17.1, and Th1 Cell Subpopulation Frequencies

**Figure 3:**
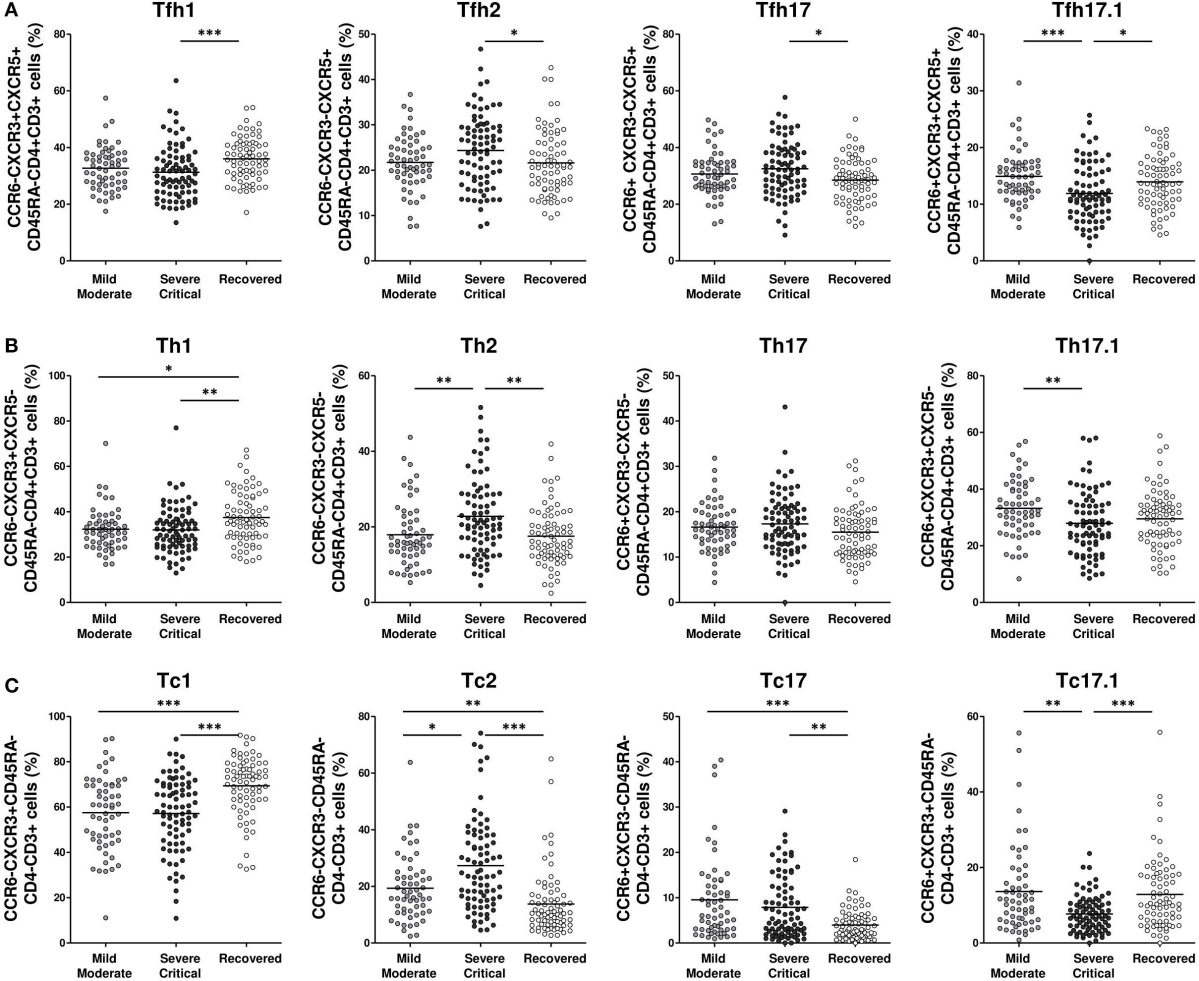
Lower Tfhl, Thl, and Tel and higher Tfh2, Th2, and Tc2 cells frequency in severe/critical COVID-19 patients. Frequency of T follicular helper cells **(A)**, T helper cells **(B)** and T cytotoxic cells **(C)** in mild/moderate (light gray circles), severe/critical (dark gray circles) and recovered (white circles) groups of patients. Each dot represents an individual patient. Data are given as mean (Kruskal–Wallis test *P-*values: *P < 0.05; **P < 0.01; ***P < 0.001). Tfh, T follicular helper; Th, T helper; Tc, T cytotoxic.

Severe/critical patients showed a significantly lower proportion of Tfh1 cells compared with recovered patients (*p* < 0.001), and of Tfh17.1 cells compared with mild/moderate (*p* < 0.001) and recovered groups (*p* < 0.05). Conversely, the percentages of Tfh2 and Tfh17 cells were significantly higher in severe/critical patients compared with recovered patients (both *p*-values < 0.05) (Figure 3A).

Non-follicular Th2 CD4 cell subpopulation frequencies were significantly higher in severe/critical patients than in mild/moderate or recovered patients (both *p*-values < 0.01). Th17.1 cell frequencies were lower in severe/critical patients than in their mild/moderate counterparts (*p* < 0.01) (Figure 3B). Th1 CD4 cell frequencies were lower in all hospitalized patients than in recovered patients (*p* < 0.01 for severe/critical and *p* < 0.05 for mild/moderate), while no differences between groups were detected for the Th17 CD4 cell subpopulation.

Differences were more pronounced in the effector CD8 subpopulations. In our cohort, all hospitalized patients had lower T cytotoxic (Tc)1 cell frequencies (both *p*-values < 0.001), and higher Tc2 and Tc17 cell frequencies than recovered patients (*p* < 0.001, *p* < 0.01 for severe/critical and *p* < 0.01, *p* < 0.001 for mild/moderate). Severe/critical patients had lower Tc17.1 frequencies than mild/moderate (*p* < 0.01) and recovered patients (*p* < 0.001) (Figure 3C).

### Severe/Critical COVID-19 Patients Had Lower Frequencies of Memory and Double-Negative B Cells and Increased Frequencies of Naïve and Plasma Cells

**Figure 4:**
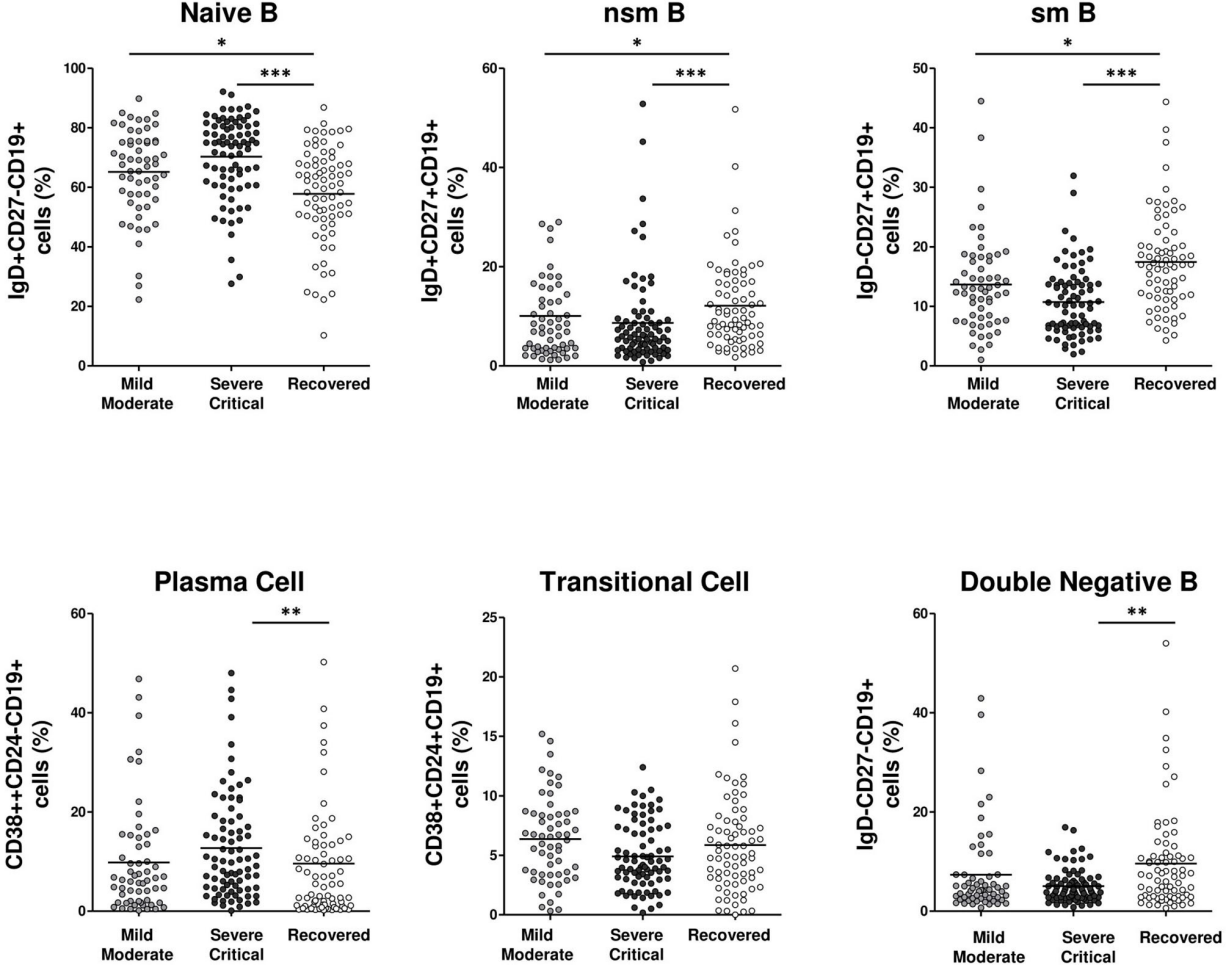
Lower memory and “double negative” and higher naive and plasma B cells frequency in severe/critical COVID-19 patients. Frequency of peripheral blood B cell populations in mild/moderate (light gray circles), severe/critical (dark gray circles) and recovered (white circles) groups of patients. Each dot represents an individual patient. Data are given as mean (Kruskal–Wallis test *P-*values: *P < 0.05; **P < 0.01; ***P < 0.001). nsm B, non-switch memory B; smB, switch memory B.

Although no differences were found in the frequency of total CD19 B cells between hospitalized and recovered patients (Figure 1B), we aimed to study if there were differences in the B cell subpopulations. All hospitalized patients had significantly higher frequencies of naïve B cells (*p* < 0.001 and *p* < 0.05) and lower frequencies of non-switched and switched memory B cells than recovered patients, although differences were more pronounced for severe/critical patients (both *p* < 0.001 for severe/critical, *p* < 0.05 for mild/moderate). However, plasma cell frequencies were significantly increased only in severe/critical patients compared with recovered patients (*p* < 0.01) (Figure 4). No differences were found in transitional B cell frequencies, while a lower percentage of double-negative B cells was described in the severe/critical group compared with the recovered patients (*p* < 0.01) (Figure 4).

Neither statistically significant correlations nor severity-adjusted correlations were found between cell subpopulations and the NEWS2 score or any laboratory marker/cytokine (data not shown).

### Prognostic Values for the Development of Severe and Critical Disease

**Figure 5:**
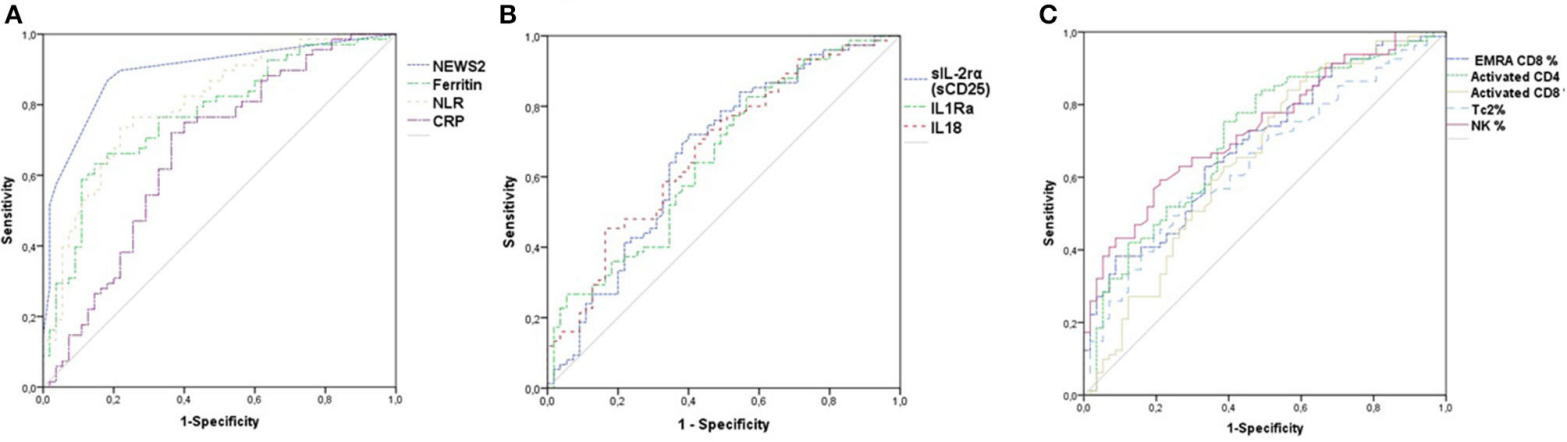
Performance of ROC curves in predicting severe/critical COVID-19 disease for News-2, Ferritin, NLR and CRP **(A)**, sCD25s, 111Ra, and IL1S **(B)** and activated CD4 and CDS, NK, Tc2 and EMRA CDS **(C)**.

A prognostic value of the clinical and biological markers was found for the NEWS2 score, ferritin, CRP, NLR, sIL-2rα (sCD25), IL-1Ra, and IL-18 as well as for activated CD4 and CD8, EMRA CD8, and Tc2 (Figure 5). Cut-off values and ORs (95% CI) for the risk of developing severe/critical COVID-19 in the univariate and multivariate analyses for NEWS2, ferritin, CRP, and NLR (model 1), for the cytokines sIL-2rα (sCD25), IL-1Ra, and IL-18 (model 2), and for activated CD4 and CD8, NK, Tc2, and EMRA CD8 (model 3) are presented in Table 2.

**Table 2:** Cut-off values and ORs (95% CI) for the risk of developing severe/critical COVID-19 in the univariate and multivariate analyses for News-2, Ferritin, CRP and NLR (model 1), for the cytokines sCD25, IL1Ra and IL18 (model 2) and for activated CD4 and CD8, NK, Tc2 and EMRA CD8 (model 3).

	**Variable**	**Cut-off**	**OR (95% CI)**	**OR (95% CI)**
			**univariate**	**multivariate**
			**model**	**model**
Model 1	News-2	2	27.7 (11.0–70.0)	41.4 (10.3–167.0)
	Ferritin (ng/mL)	583	11.8 (4.9–28.4)	16.3 (3.8–69.9)
	CRP (mg/dL)	5.3	11.0 (4.9–24.5)	2.5 (0.7–8.5)*
	NLR	3.0	4.1 (2.0–8.5)	3.5 (1.0–12.0)
Model 2	sCd25 (pg/ml)	512	3.9 (1.7–8.9)	3.3 (1.4–7.9)
	IL1Ra (pg/ml)	94.0	3.9 (1.8–8.4)	3.2 (1.4–7.3)
	IL18 (pg/ml)	125	3.1 (1.5–6.4)	2.4 (1.1–5.0)
Model 3	Activated CD4 (%)	5.1	4.6 (2.2–9.6)	2.2 (0.9–5.4)*
	Activated CD8 (%)	15.2	4.0 (1.8–8.7)	1.7 (0.7–4.4)*
	NK (%)	17.5	5.0 (2.3–10.9)	3.3 (1.4–7.8)
	Tc2 (%)	23.3	3.2 (1.6–6.7)	2.8 (1.2–6.4)
	EMRA CD8 (%)	53.5	6.2 (1.6–6.5)	2.0 (0.9–4.5)*

*NLR, Neutrophil/lymphocyte ratio; CRP, C Reactive protein; Activated CD4, CD38+HLA-DR+CD4+; Activated CD8, CD38+HLA-DR+CD8+; Tc2, CCR6-CXCR3-CD45-RA-CD4-CD3+; EMRA CD8, CD45RA+CCR7-CD8+*.

*Not statistically significant.

## Discussion

Immune response to SARS-CoV-2 resembles other antiviral responses in non-severe individuals, with a rapid activation of the innate immune response that contains the viral spread while the adaptive immune response develops. Patients who recover from the disease develop both neutralizing antibodies and virus specific memory T cells (18), indicating that both T and B cells are important players in the response. Patients unable to contain the virus in the acute phase develop a hyperinflammatory state causing a cytokine storm and exhaustion of the adaptive response that results in severe disease and eventually death. Identifying immune dysfunction patterns common to severely ill patients will allow to anticipate which patients will require Intensive Care Unit (ICU) admission and help to identify potential therapeutic targets.

In this study, we compared the immune phenotypes of 58 mild/moderate, 81 severe/critical, and 74 recovered patients. There was clear evidence of a hyperinflammatory state in the severe/critical group of patients, with higher levels of proinflammatory cytokines (IL-6, IL-18), IL-1Ra, sIL-2rα (sCD25), IL-10, ferritin, D-dimer, and complement factor C3. Severe/critical patients exhibited generalized lymphopenia but normal NK cell numbers. A decrease in CD3+ T cell frequency (explained mostly by a CD4+ decrease) and an increase in NK cells were evident in the severe/critical group. CD4+ and CD8+ T cells had an increased activated (HLA-DR+ and CD38+) and EMRA phenotype, and an increased Tfh2, Th2, and Tc2, and decreased Tfh17.1, Th17.1, and Tc17.1 phenotype in severe/critical patients. Severe/critical patients had significantly higher frequencies of naïve and plasma B cells and lower frequencies of non-switched and switched memory B cells than recovered patients.

Hyperferritinaemia and high sIL-2rα (sCD25) levels are included in the diagnostic criteria of haemophagocytic lymphohistiocytosis and have been related to severity and mortality in COVID-19 in several studies (11, 19, 20) and in a meta-analysis (21). An association between increased in-hospital death risk and D-dimer >1 μg/mL has also been found (22, 23). High levels of the complement component C3, point of convergence of the three complement activation pathways, may also be related to the hyperinflammatory state, as described in acute infections and metabolic syndrome (24, 25). Therapeutic C3-targeted interventions are under investigation in COVID-19 (26).

Proinflammatory cytokines, including IL-6, IL-7, IL-8, TNF-α and others, and the anti-inflammatory cytokine IL-10 have been widely associated to disease severity and death in COVID-19 (7, 11, 27–30), but their use and usefulness in routine clinical practice is still debated. The elevated serum IL-1Ra levels found in our severe/critical patients probably reflect elevated levels of IL-1β that remain undetected due to the short half-life of this cytokine. In our study, IL-18 levels were significantly elevated in severe/critical patients compared with mild/moderate patients. IL-18, a surrogate marker of inflammasome activation, has recently been described as a promising marker of COVID-19 severity (31–33). In response to SARS-CoV-2, the NLRP3 inflammasome activates caspase-1, which cleaves gasdermin D and the precursor cytokines pro-IL-1β and pro-IL-18, initiating pyroptosis and maturation of IL-1β and IL-18. Most patients who develop severe/critical COVID-19 have risk factors (obesity, diabetes, heart disease, hypertension, or aging) characterized by chronic inflammasome activation that may account for the progression to respiratory failure in the context of chronic inflammation (34). However, we did not find higher levels of IL-18 in severe/critical patients with any of the above risk factors compared with those without them (data not shown), which supports its role as an independent marker of severity. New therapies that inhibit NLRP3 are under investigation, and phase II clinical trials are ongoing (34).

In our study and others (6, 11, 35), patients who developed severe/critical disease had a significantly elevated NLR related to lymphopenia and these markers have been included in previously proposed severity scores (13, 14). The pathogenesis of peripheral blood lymphopenia has not yet been fully elucidated, and many factors seem to be implicated: apoptosis (36), pyroptosis mediated by inflammasome activation (32), direct cytopathic effect of the virus, and lymphocyte infiltration and sequestration in the lungs.

We observed a decrease in the CD3+ T cell frequency (explained mostly by a CD4+ decrease) and an NK cell increase in the severe/critical group. CD4+ and CD8+ T cells showed an increased activated (HLA-DR+ and CD38+) and EMRA phenotype in severe/critical patients and higher frequencies of naïve and plasma B cells with lower frequencies of non-switched and switched memory B cells. Robust CD4 activation and proliferation, decreased T follicular cells, increased plasmablast response, and exhausted CD8 T cells has been described as a phenotype associated with severe disease, while classical CD8 response with low CD4 and plasmablast response represents a more favorable immune landscape (9). The increase in HLA-DR and CD38 expression associated with activation of CD8 and CD4 T cells in critical COVID-19 patients (37) has already been found in association with an increased frequency of terminally differentiated EMRA CD4+ and CD8+ cells. Activated T cells can be recruited to infection sites to fight viruses by secreting cytokines, promoting cytotoxicity but also pathogenicity and causing severe disease. The presence of highly activated T cells in COVID-19 patients has been related to a senescent phenotype and the expression of inhibitory receptors, suggesting that these cells are exhausted. However, Shahbaz et al. have demonstrated decreased but highly activated T cells in COVID-19 patients. In these cells, upregulation of coinhibitory receptors was accompanied by an increased cytokine production capacity, reflecting an activated rather than exhausted phenotype (38). Treg cell frequency was decreased in both groups of hospitalized patients compared with recovered patients, although the difference was only statistically significant for severe/critical patients. Thus, this scenario of immune hyperactivation described in our severe/critical patients could be related to overstimulation secondary to the hyperinflammatory state or a lack of regulation by Treg cells.

Our major contribution is the comprehensive characterization of helper, follicular, and Tc subpopulations performed in this study. A variety of CD4+ T cell subsets can participate in a humoral immune response through common patterns of cytokine production (39). A decrease in the percentage of Th1 cells was already evident in the mild/moderate forms of the disease and was accompanied by a decrease of the Th17.1 and an increase of the Th2 subpopulations in the severe/critical form. A negative clinical course in COVID-19 patients has been related to an underrepresentation of the Th1 subset (15). Th1 lymphocytes are essential for the immune response to intracellular microorganisms and viruses through the secretion of IL-2 and IFN-γ which activates CD8+ cytotoxic cells that destroy virus infected cells (40). Moreover, antibody response to SARS-CoV-2 correlates with the Th1 response (41). A higher percentage of senescent Th2 cells has been found in patients who died than in those who survived, and it has been considered as an independent risk factor for death (42). Roncati et al. found evidence of Th2 response in COVID-19 patients requiring intensive care (43). We did not find differences in the Th17 subpopulation. There is controversy on the role of Th17 response in COVID-19. Evidence suggests that it plays an important role in the pathogenesis of COVID-19 through the release of IL-17 and GM-CSF, promotion of neutrophil migration, induction of Th2 and inhibition of Th1 differentiation, and downregulation of Treg response (44). However, efficacy of secukinumab, an anti-IL-17 monoclonal antibody, was not demonstrated in a phase II controlled trial (45). Follicular T cells are essential for B cell proliferation, survival, differentiation, and antibody responses. Eisenbarth et al. in their opinion article, propose a uniform nomenclature for follicular T cells. They emphasize the plasticity of the Tfh subset and its capacity for interconversion with other T cell subsets (39). We also found an altered distribution of Tfh populations in our severe/critical group. There was an analogous increase of both Tfh2 and Tfh17 frequency together with a decrease in Tfh1 frequency in severe/critical patients compared with recovered patients. Moreover, there was a decrease in Tfh17.1 cells in severe/critical patients compared with mild/moderate and recovered patients. We also found a strikingly altered distribution of Tc populations in our COVID-19 patients. Both hospitalized groups showed a high Tc2 and Tc17 and a low Tc1 cell frequency relative to recovered patients. However, there was a decrease in the proportion of Th17.1 cells only in severe/critical patients compared with mild/moderate and recovered patients. Considering all three T cell population responses as a whole, we detected an underrepresentation of T1 and T17.1 response and an overrepresentation of Th2 and Th17 in severe/critical patients.

Although no differences were found in the frequencies of total B lymphocytes between the different groups of patients, subpopulation distribution was strikingly different. Previous studies have shown a correlation between the frequency of memory B cells, both non-switched and switched, and disease resolution (46). A decrease in both non-switched and switched memory B cells and an increase in naïve B cells accompanied by a corresponding increase in the naïve subpopulations was a common finding in our hospitalized patients, although it was more pronounced in the severe/critical group. This may represent exhaustion and overactivation of these subpopulations (9, 47–49). A high proportion of plasma cells was a characteristic of the severe/critical group. The mechanism and relevance of the plasma cell increase and the relationship with severity is unclear although it has been related to the early production of anti-SARS-CoV-2 antibodies (50, 51). Several studies have demonstrated a correlation between the frequency of plasmablasts and the severity of the disease in other viral infections (52). In our case, the percentage of double-negative B cells was decreased in the severe/critical group. Although the function of the different subpopulations of double-negative B cells has not been elucidated, some authors have described altered distributions of subpopulations of these cells in COVID-19 patients related to severity (53). Thus, the severity of the disease is related to a progressive decrease in memory B cell populations that characteristically culminates in an increase of plasma cells in the more severely ill patients. These populations return to baseline levels as patients recover.

Our study has some important limitations. It was conducted in a single center with a limited number of patients and, since only peripheral blood was analyzed, no correlations with immunological changes in bronchoalveolar lavage fluid were determined.

In conclusion, the determination of several cytokines, notably IL-18 and sIL-2rα (sCD25), and the flow-cytometry analysis of activated CD4, CD8, NK, Tc2 CD8, and EMRA CD8 cells on admission may have a prognostic value, but it does not replace other laboratory parameters such as ferritin, CRP, or NLR, nor the easy-to-perform clinical NEWS2 score. However, our results contribute to the understanding of COVID-19 immunopathology and add relevant information on the involvement of the adaptive immune system in the physiopathology of severe forms of COVID-19. Evaluating the immunophenotype of relevant peripheral blood subpopulations may help to identify those patients at risk of a critical disease course and apply early therapeutic measures to control disease severity. It may also provide clues for specific interventions to restore the balance of the adaptive immune system subpopulations involved in the severity of the disease.

## Data Availability Statement

The raw data supporting the conclusions of this article will be made available by the authors, without undue reservation.

## Ethics Statement

The study was approved by the local Ethics Committee (Comité Ético de Investigación Clínica Illes Balears no. IB 4169/20 PI). The patients/participants provided their written informed consent to participate in this study.

## Author Contributions

MG-G, MB-R, and JF: conception and design of the work, acquisition, analysis and interpretation of data, and drafting the work and revising it. JP, NM-P, MR, and JM: conception and design of the work and revising the manuscript critically for important intellectual content. AR, AI, IL-A, AF-C, and AB: acquisition and analysis of the data and revising the manuscript critically for important intellectual content. All authors provided approval for publication of the content and agree to be accountable for all aspects of the work in ensuring that questions related to the accuracy or integrity of any part of the work are appropriately investigated and resolved. All authors contributed to the article and approved the submitted version.

## Funding

This work was supported by a grant from Instituto Salud Carlos III [grant number COV20/00943]. Publishing fees were supported by Fundació Institut d'Investigació Sanitària Illes Balears – IdISBa.

## Conflict of Interest

The authors declare that the research was conducted in the absence of any commercial or financial relationships that could be construed as a potential conflict of interest.

## Publisher's Note

All claims expressed in this article are solely those of the authors and do not necessarily represent those of their affiliated organizations, or those of the publisher, the editors and the reviewers. Any product that may be evaluated in this article, or claim that may be made by its manufacturer, is not guaranteed or endorsed by the publisher.

## Abbreviations

COVID-19: coronavirus disease 2019
SARS-CoV-2: severe acute respiratory syndrome coronavirus 2
T reg: T regulatory
Tfh: T follicular helper
Th: T helper
Tc: T cytotoxic
NK: natural killer
sIL-2rα: serum soluble IL-2 receptor Alpha
IL-1Ra: IL-1 receptor antagonist
IFN: interferon
TNF-α: tumor necrosis factor alpha
NLR: neutrophil/lymphocyte ratio
EMRA: effector memory T cells reexpressing CD45RA.
